# **Microbiota** [mī′′-krō-bī′-ō-′tә], **microbiome** [mī′′-krō-bī′-ōm]

**DOI:** 10.3201/eid3008.230677

**Published:** 2024-08

**Authors:** Julia Memrava Cabrera, Daniel F.M. Monte

**Affiliations:** Sao Paulo State University, Jaboticabal, Brazil (J.M. Cabrera);; Federal University of Paraiba, Areia, Brazil (D.F.M. Monte)

**Keywords:** Etymologia, microbiota, microbiome, bacteria, viruses, fungi, microbial communities, microorganisms, Joshua Lederberg

## Microbiota [mī′′-krō-bī′-ō-′tә], microbiome [mī′′-krō-bī′-ōm]

From the Greek *micro-* (small) and -*bios* (life), microbiota was coined in the late 19th Century to denote the microorganisms residing in a specific environment. During the 20th Century, microbiota became more specifically associated with the microorganisms inhabiting the human body. Today, the term encompasses the collective genetic material of microorganisms, spanning viruses, archaea, bacteria, and fungi, and the intricate ecosystems of microorganisms, including commensal, symbiotic, and pathogenic ones, that exist within or on the human body or other environmental niches. Exploring microbiota and its implications in various aspects has rapidly gained momentum as a dynamic field of research.

The term microbiome was defined by Whipps and colleagues in 1988 as the collective genomes of microorganisms. However, Joshua Lederberg (Figure), a US molecular biologist, played a pivotal role in coining the term as we know it today. His journey from a precocious young scientist to a Nobel laureate and advocate for ethical science reflects the interconnectedness of language, curiosity, and scientific discovery. Lederberg’s fascination with science also extended to writing science fiction, using the genre to explore complex scientific concepts through imaginative storytelling. In fact, microbiome is a combination of *microbe* and *biome* (*bi-* [life] + *-ome* [mass]) to describe the microbial ecosystem, which encompasses not only genomes but also the broader microbial environment. Microbiome, born from the fusion of linguistic roots and a thirst for knowledge, continues to shape our understanding of the microbial world and its profound impact on human health and biology.

**Figure:**
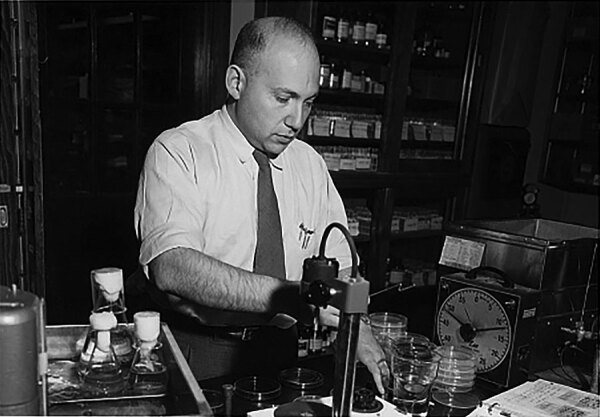
Molecular biologist and Nobel laureate Joshua Lederberg in his laboratory in Wisconsin, 1958. Dr. Lederberg played a pivotal role in coining the term microbiome as we know it today. Public domain image from the National Library of Medicine.
